# Supplementary material for: Batch correction for large-scale mass spectrometry imaging experiments
Source: Bioinformatics. 2026 Jun 4;42(6):btag360. doi: 10.1093/bioinformatics/btag360 (PMC13281927; doi:10.1093/bioinformatics/btag360)
Supplement: btag360_Supplementary_Data [file btag360_supplementary_data.docx]

Supplementary material for:

Title: **Batch correction for large-scale mass spectrometry imaging experiments**

Authors: Andreas A. Sparre and Ole N. Jensen.

Affiliation: Department of Biochemistry and Molecular Biology, University of Southern Denmark, DK-5230 Odense M, Denmark.

**Supplementary**

**S1 UMAPs of all pixels after CCA and fastMNN correction**

**CCA UMAP – all pixels colored by biological origin**


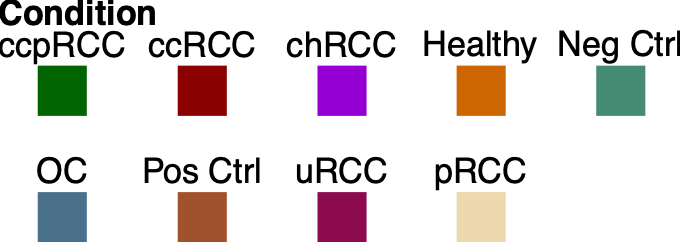
**
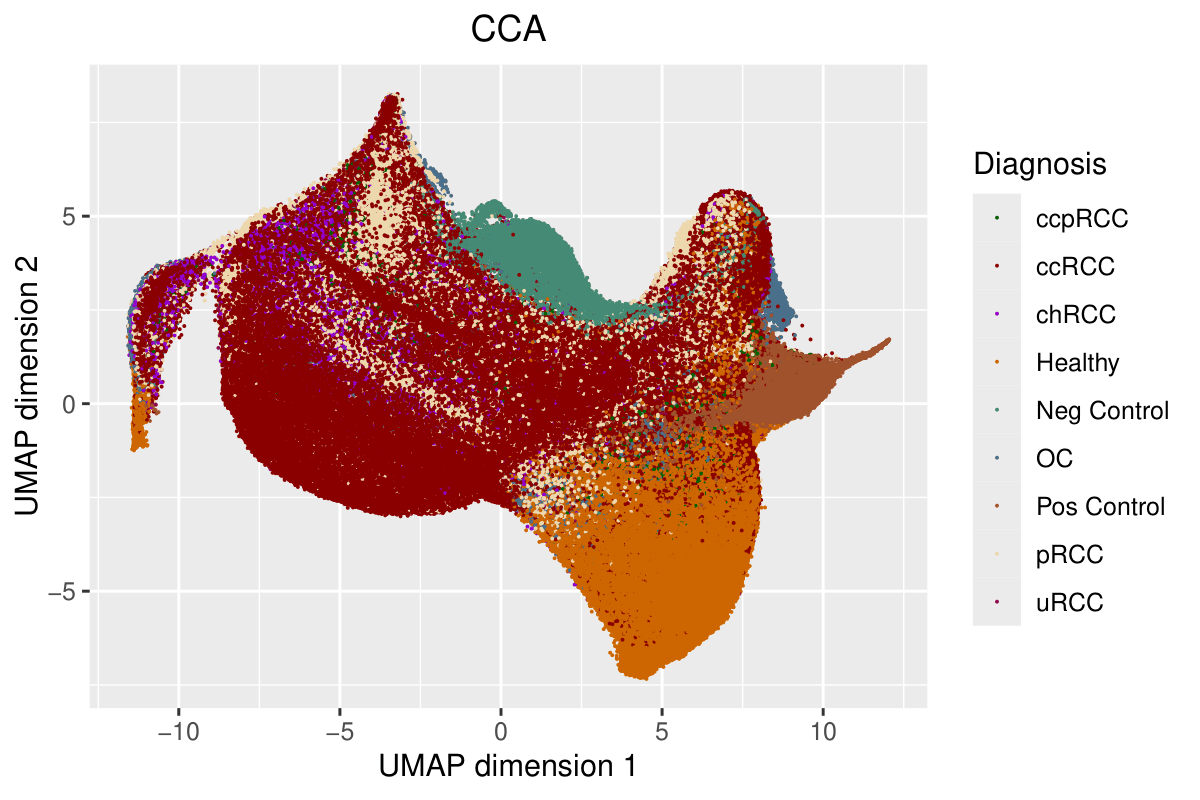
**

**CCA UMAP – all pixels colored by batch**


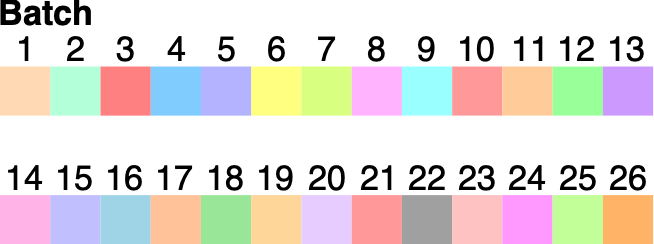
**
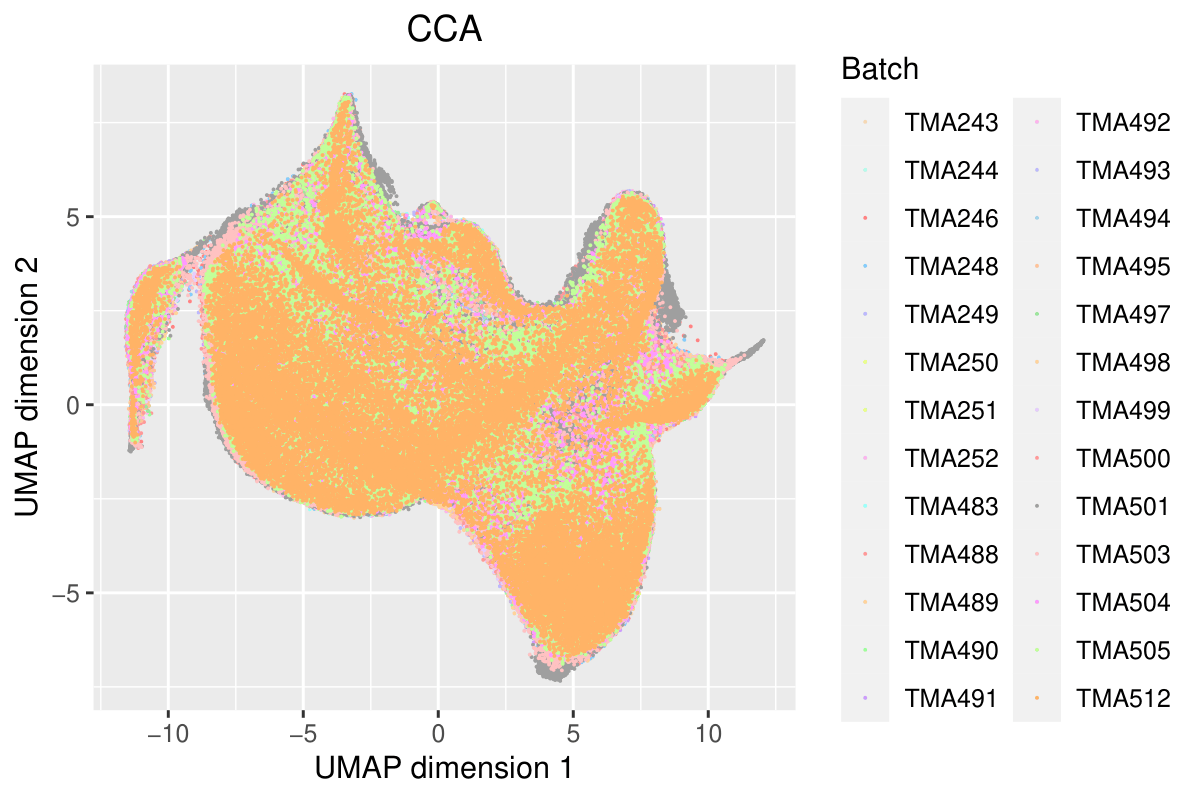
**

**fastMNN UMAP – all pixels colored by biological origin**


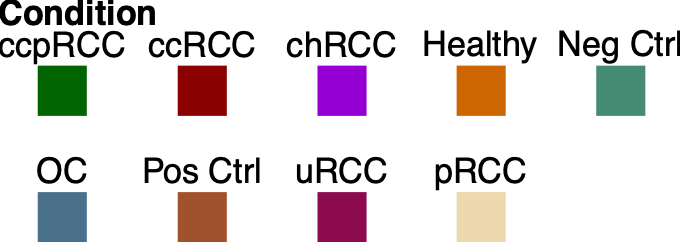
**
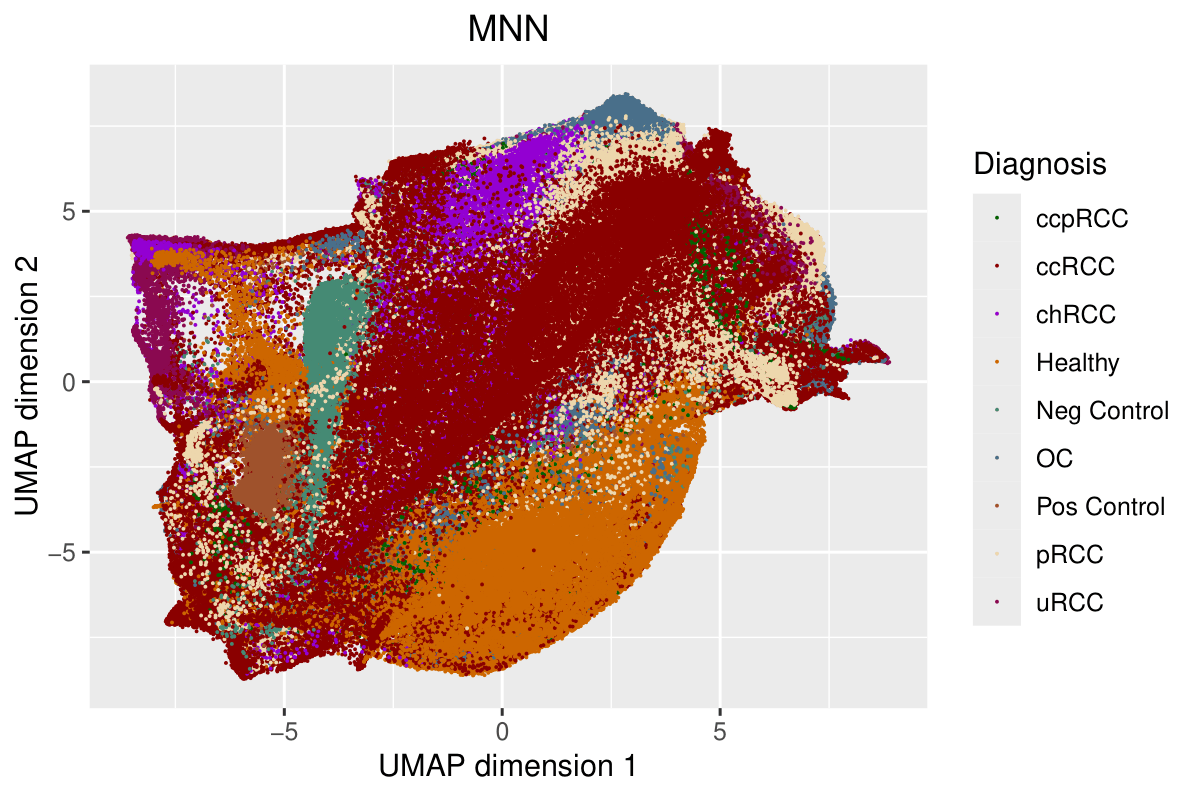
**

**fastMNN UMAP – all pixels colored by batch**


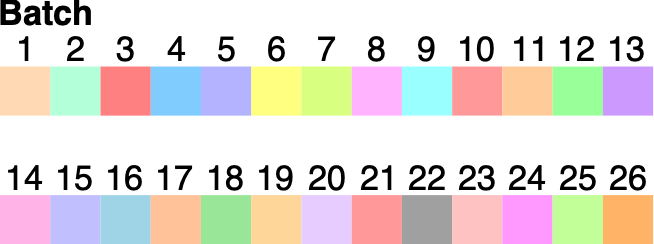
**
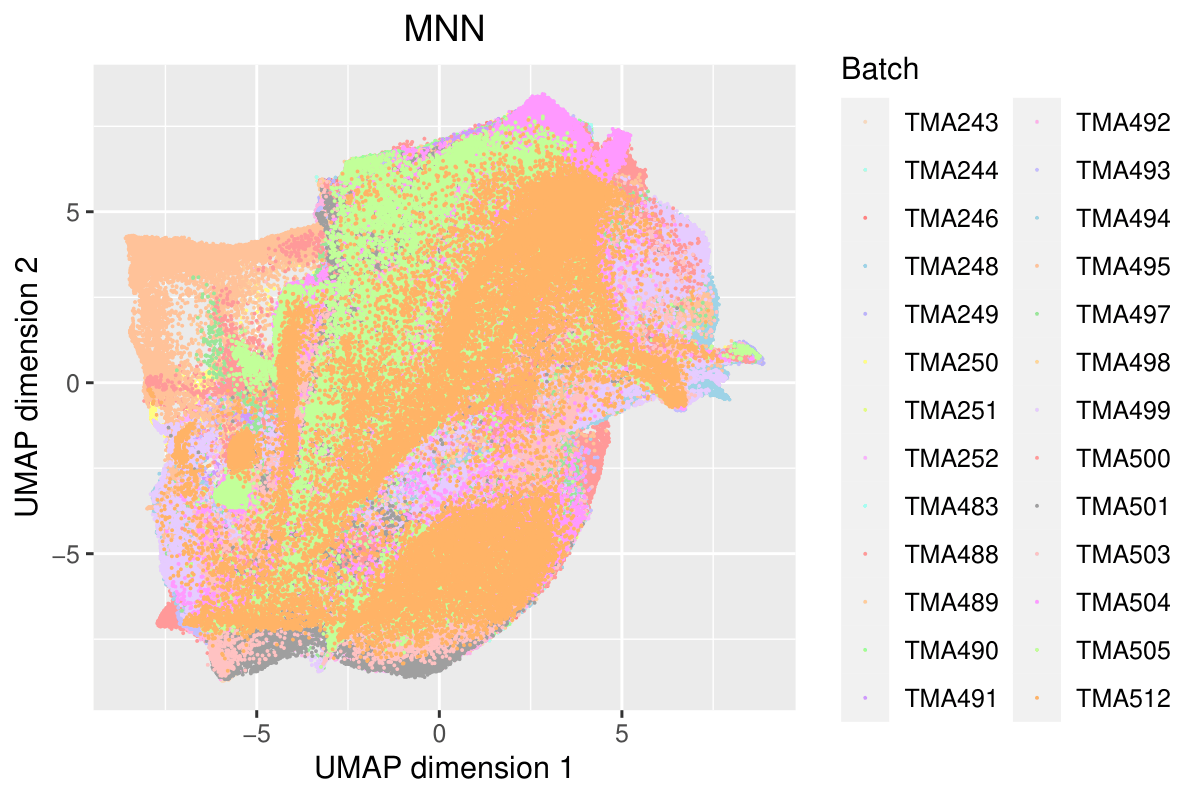
**

**S2 – Ion images of selected features**

**
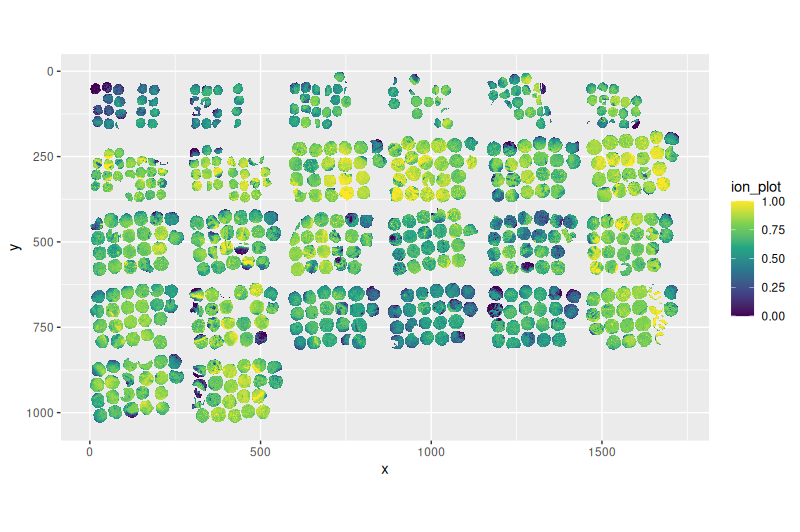
Ion image of *m/z* 1198.7110 – uncorrected**

**
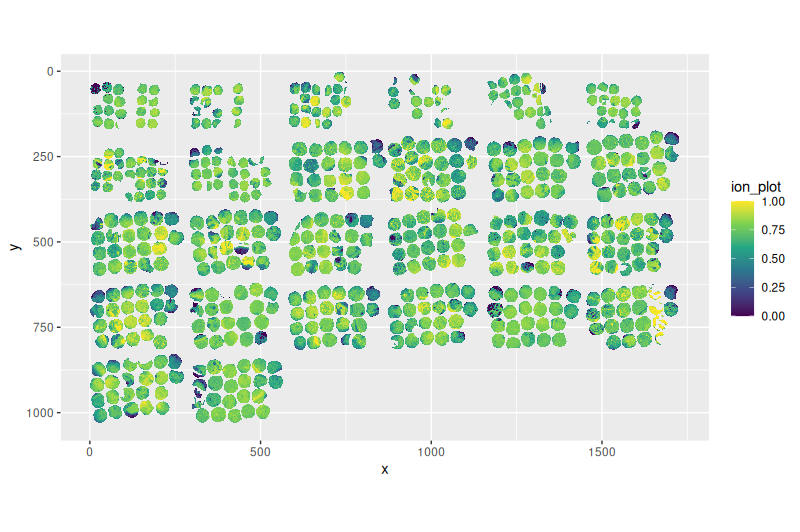
Ion image of *m/z* 1198.7110 – after comBAT correction**

**Ion image of *m/z* 2676.2669 – uncorrected**

**
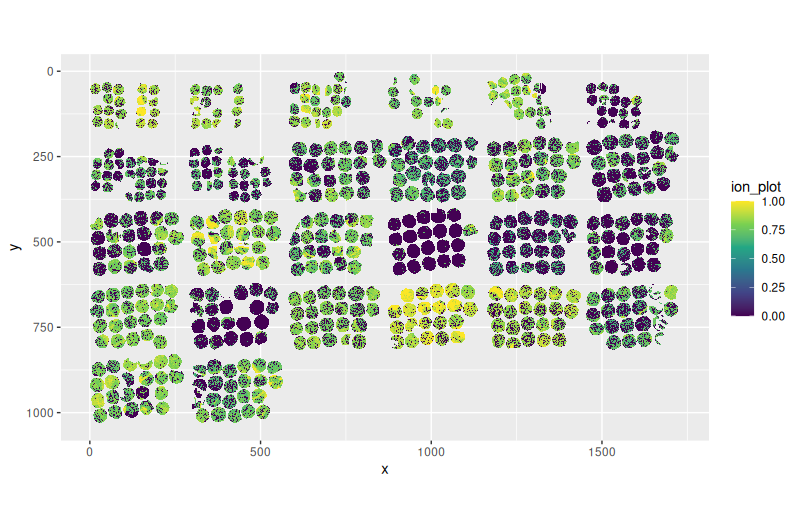
**

**Ion image of *m/z* 2676.2669 – after comBAT correction**

**
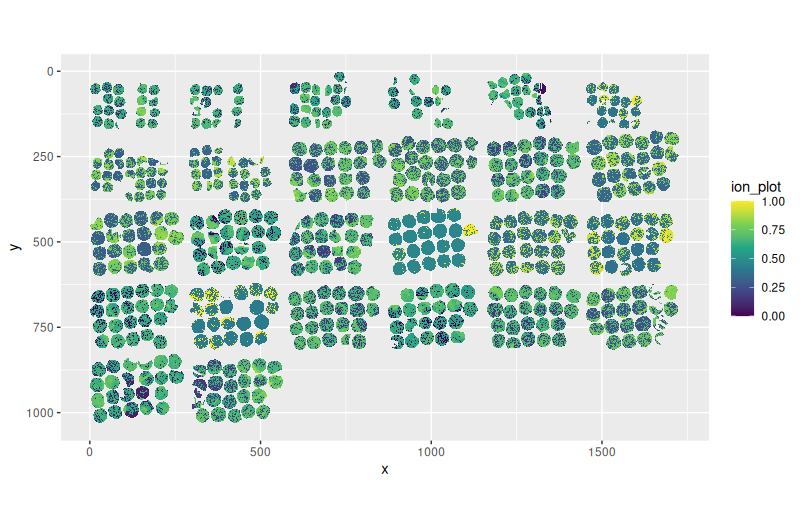
**
